# Supplementary figures and images for: RPSLearner: A novel approach based on random projection and deep stacking learning for categorizing NSCLC
Source: bioRxiv. 2025 May 7:2025.05.01.651699. Preprint. [Version 1] doi: 10.1101/2025.05.01.651699 (PMC12247899; doi:10.1101/2025.05.01.651699)

a)

## Subtype percentage

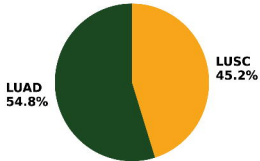

b)

## Project percentage

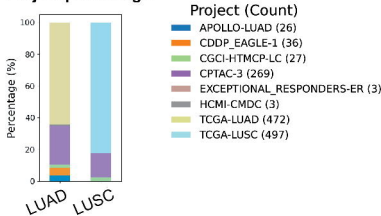

a)

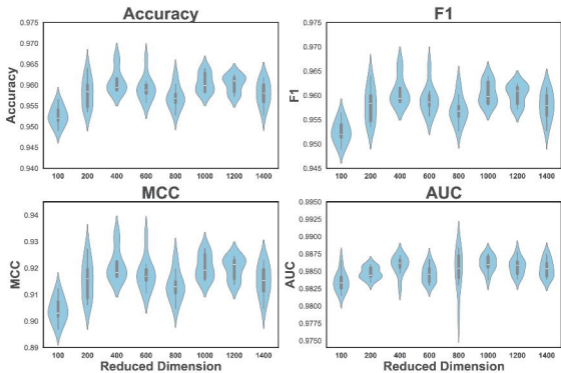

b)

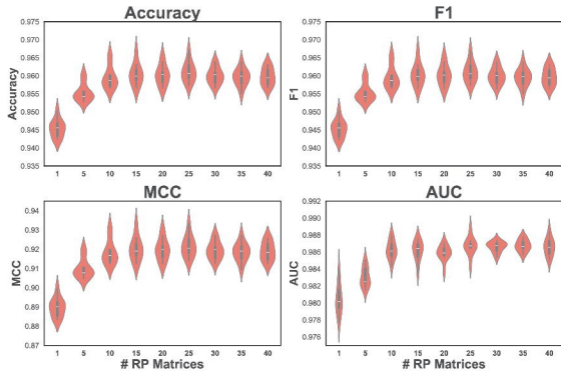

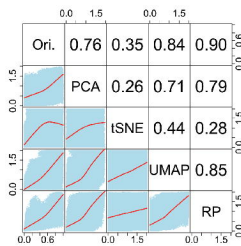

Dim 20

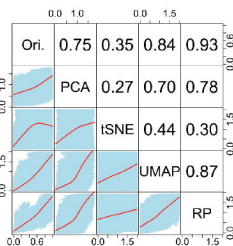

Dim 80

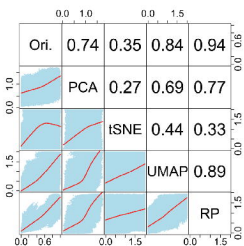

Dim 140

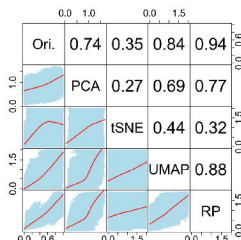

Dim 200

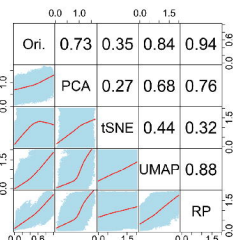

Dim 260

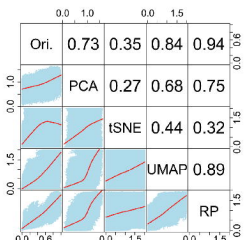

Dim 320

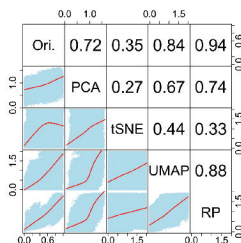

Dim 380

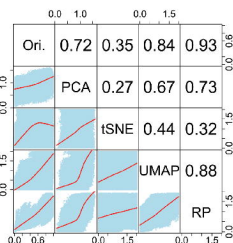

Dim 440

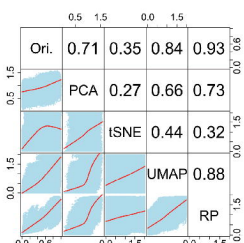

Dim 500

Supplement: Supplement 1 [file NIHPP2025.05.01.651699v1-supplement-1.pdf]
